# Fisherfolk's Perception of and Attitude to Solid Waste Disposal: Implications for Health, Aquatic Resources, and Sustainable Development

**DOI:** 10.1155/2021/8853669

**Published:** 2021-04-15

**Authors:** Justice Mensah

**Affiliations:** Directorate of Academic Planning and Quality Assurance, University of Cape Coast, Cape Coast, Ghana

## Abstract

The study explored fisherfolk's perception and attitude in regard to solid waste disposal and the implications of these for public health, aquatic resources, and sustainable development (SD) in a Ghanaian fishing community. Qualitative data were obtained from 37 purposively targeted participants comprising 34 fisherfolk, an environmental health expert, a water and sanitation expert, and a fisheries and aquatic sciences expert through in-depth interviews and focus group discussions. Data were analysed thematically guided by the most significant stories. While the fisherfolk perceived waste as useless and a nuisance, the experts saw it as a nuisance and/or resource. The fisherfolk did not sort their waste in line with best practices, nor were they willing to pay for waste collection services, citing poverty as the main reason. While most fisherfolk disposed of their waste into bins as approved, others did so onto the street, into the drains, lagoon, and sea. While the fisherfolk were aware that indiscriminate waste disposal led to diseases such as malaria and cholera, their perceptions of the effect of the same on aquatic resources were mixed. The fisherfolk's conception and perception of waste led to attitudes, behaviours, and practices that polluted the environment (air, land, and water resources), resulting in public health challenges, a threat to navigation, fish population, and other biodiversity, which were inimical to livelihoods and sustainable development. The government and municipal authority should collaborate with the experts in environmental health, water and sanitation, fisheries and aquatic sciences, and traditional authorities to sensitise the fisherfolk on the sustainability implications of unapproved solid waste disposal practices to change their attitude for the better. They should also provide more waste disposal infrastructure and enforce the laws to ensure compliance with best practice for sustainable development. The study supports the compatibility and mutuality between Sustainable Development (SDG) 6 on water, sanitation, and hygiene and SDG 14 regarding sustainable use of the oceans, seas, and marine resources.

## 1. Introduction

Waste management has been an issue of global concern throughout history and continues to be so today. Given the importance and complexity of the issue, the global leaders considered it more seriously in the health and environmental management targets of the Millennium Development Goals (MDGs) and the Sustainable Development Goals (SDGs) in the years 2000 and 2015, respectively. Similarly, the United Nations (UN), through the World Summit on Sustainable Development (SD) in Johannesburg in 2002, set aside the year 2008 as the “International Year of Sanitation” to draw attention to the importance of environmental sanitation and hygiene for SD. Despite all these, indiscriminately disposed wastes continue to define the physical environment of many countries around the globe, most especially, developing countries.

Kalumbi et al. [1] have highlighted the growing threats to aquatic resources and public health from environmental degradation caused principally by improper waste disposal practices in Africa and other parts of the world. The threats are incompatible not only with the SDG 6 on sanitation and hygiene but also with SDG 14, which emphasises the conservation and sustainable use of the oceans, seas, and marine resources. Individuals, households, institutions, municipalities, and industries are the key responsible entities for the generation and disposal of the wastes. However, one industry whose waste disposal practices have not attracted the research prominence it deserves, especially in developing countries, is the fishing industry [2–4], despite the threats posed by the industry's disposal activities to sustainable livelihoods.

In Ghana, the fishing industry contributes to Gross Domestic Product (GDP) and provides livelihoods for many people, particularly the poor coastal fisherfolk. However, poor environmental sanitation in the fisherfolk's workplace environment threatens their health and the aquatic resources that are important for their livelihoods. The poor environmental sanitation is, in part, attributed to the fisherfolk's perception of waste and attitude to waste disposal, especially solid waste [3]. While many studies have been done in the area of solid waste management practices in Ghana, a limited number of them have been directed at coastal fisherfolk's workplace environment. The limited studies have paid little attention to the perception and attitude regarding waste disposal, although extant theoretical literature [5, 6] indicates that these are key factors in safeguarding public health, aquatic resources, and livelihoods, especially in the coastal poor communities.

Elmina is one of the most popular coastal communities in Ghana, where the indigenous people depend mostly on fishing-related economic activities for livelihood. However, the physical environment in which the fisherfolk ply their trade is engulfed in filth as a result of the people's poor waste disposal attitude and practices. The fisherfolk engage in navigation on the lagoon and sea for fish, mending of fishing nets, canoes, boats, and gears, and preservation (smoking, salting, drying, and freezing) and sale of fish. In the course of these activities, wastes are generated that need to be disposed of or managed. As indicated by Boopendranath [2], sources of wastes generated in capture fisheries include by-catch discards; onboard processing wastes; metal and plastic wastes due to abandoned, lost, or discarded fishing gear; and food materials, bilges, and other wastes from vessel operations. Apart from posing public health threats, the wastes that eventually get into the lagoon, sea, and ocean could also have implications for the aquatic resources. Despite its potential implications for livelihoods and SD, the fisherfolk appeared unconcerned about this phenomenon. This provoked an investigation as to the fisherfolk's perception of and attitude to solid waste disposal and the implication of these for public health and aquatic resources that constitute their natural capital for livelihood. The objective of the study, therefore, was to explore local fisherfolk's perception of and attitude to solid waste disposal and the implications of these for public health, aquatic resources, and sustainable development.

To set the tone for the study, literature was reviewed on key conceptual issues. These were waste, perception, attitude, and their implications for health, aquatic resources, and sustainable development. An exploration into literature showed that waste defies precise definition but the various descriptions and definitions of the concept suggest that it is an abandoned material that has no consumption value to the one discarding it. Waste can be hazardous or nonhazardous depending on its location and toxic concentration; therefore, it needs to be properly disposed of or managed. Waste can be disposed of in many ways, including burying, recycling, incineration (burning), composting landfill, and indiscriminate dumping such as dumping it on the street and into the water bodies [5]. UNEP [7] expresses concern about waste abandoned in the marine and coastal environment. This is because marine litter could cause direct or indirect damage to marine ecosystems as well as human activities such as fishing and aquaculture, shipping, tourism, and recreational activities Werner et al. [8].

Perception and attitude influence waste disposal practices. Barnhart [9] and Oyedotun et al. [10] observe that perception relates to the state of being aware of something through the senses: sight, hearing, taste, smell, and touch/feeling. Though it may be subjective, perception enables people to react to a situation or phenomenon since it influences understanding, interpretation, impression, and viewpoint regarding how individuals see things, which suggest awareness, knowledge, belief, and expectations. As noted by Gyankumah [11], efforts to address solid waste disposal challenges in developing countries have failed due to the negative perception people have regarding solid waste disposal.

Attitude is an enduring predisposition towards a particular aspect of one's environment [12]. It consists of three basic components, namely, perception which relates to emotional impression, cognition which relates to thought, and a tendency to act. In Ajzen's view [13], attitude is influenced by the subjective belief that an action will produce a specific result. This hinges on the individual's evaluation of the phenomenon. In other words, people's positive attitude towards a phenomenon will strengthen their intention to perform a specific action [14,15]. Waste disposal is linked to people's attitudes towards littering, interest in waste reduction, and minimisation. It is argued [16] that people's attitudes influence their behaviour towards waste disposal. Where the waste disposal attitude and behaviour are negative, the disposal practices could have negative environmental, social, and economic impacts, thereby affecting SD. The reverse socioeconomic and environmental impacts are registered where the waste disposal attitude and behaviour are positive, resulting in eventual positive effects on SD.

## 2. Study Setting and Methods

**Figure 1:**
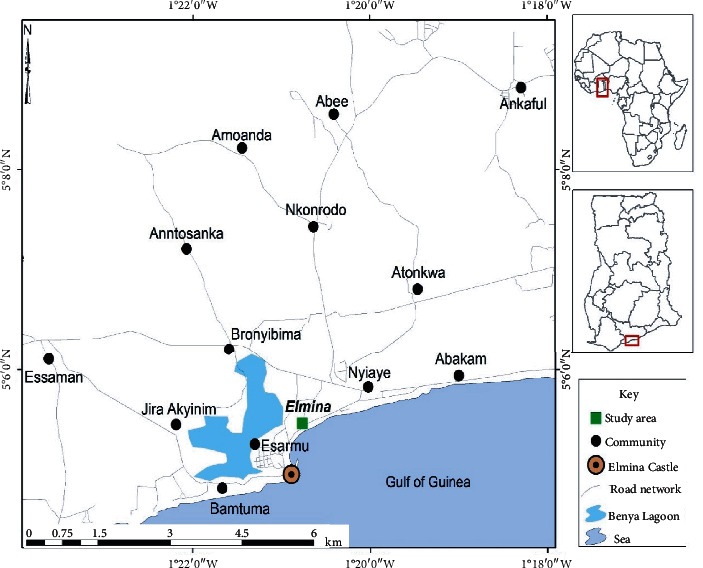
Map of Ghana showing the geographical location of the study area (source: Cartography Unit, University of Cape Coast, Ghana).

The study was conducted at Elmina (Figure 1), a coastal town in the Central Region of Ghana. Elmina is one of the most popular fishing communities in Ghana. The indigenous people are poor fisherfolk and indulge in various forms of improper sanitation practices which could impact their health and the aquatic resources—their greatest livelihood assets.

The research adopted the qualitative case study design. The rationale was to collect data using a naturalistic approach, focusing on the meaning actors ascribe to the phenomenon of waste disposal. Qualitative research involves an iterative process in which improved understanding of the scientific community is achieved by making significant contributions resulting from getting closer to the phenomenon studied [17]. The study targeted fisherfolk close to the sea and lagoon at Elmina, as the primary respondents. It used specific confines of Elmina—immediate vicinity of the sea and the lagoon—since that was where the typical fisherfolk in the community lived. Experts in water and sanitation, environmental health (sanitation), and fisheries and aquatic sciences were also involved as participants. The rationale was to compare the perspectives of the experts and fisherfolk. In-depth interview guides (IDIs) and focus group discussion (FGD) guides were developed for the study. The instruments contained items on perceptions of waste disposal, attitude to waste disposal at the fisherfolk's workplace, and implications of waste disposal for health, aquatic resources, and sustainable development.  Table 1 presents the profile of respondents, the data collection approach, and the instruments used.

Some questions varied across the instruments, while others were similar to make sure that the central themes were covered while providing a meaningful basis for comparison. The instruments were vetted for content validity and quality assurance at the Institute for Development Studies (IDS) of the University of Cape Coast, Ghana. The Institute's approval letter with reference number IDS/40/Vol.4/194 also served as an introductory document for data collection in addition to the informed consent forms designed for the respondents.

Three research assistants were recruited to assist with data collection and analysis. One of the assistants had a master's degree in sociology, another had a master's degree in geography, and the third had a first degree in fisheries and aquatic sciences. These were trained for 16 days on the content of the instrument, data collection, translation from English into local languages, research ethics, and data analysis. The training on data analysis centred on coding, recoding, developing themes, data grouping, and re-grouping based on similarities and differences among the themes. The instruments were pretested at Ankwanda, a fishing community in the Komenda-Edina-Eguafo-Abrem Municipality in the Central Region of Ghana. During the pretest, each of the data collectors conducted one interview while all of them (together) conducted two FGDs, one with fishermen and another with fish preservers. Each FGD was composed of 12 participants. The instruments were in English but were translated on-site into the respondents' vernacular (local language). Led by the researcher, the research assistants transcribed, analysed, and discussed the pretest data for common understanding to ensure there was no challenge with translation and interpretation which could compromise methodological accuracy. Lessons learned from the pretest of the instruments were used to revise the instruments for the actual data collection (main fieldwork). In this regard, ambiguous questions were reframed while the translation of technical terms was revisited to make issues clearer to the participants during the actual data collection.

The actual data collection took place from 27^th^ January 2017 to 23^rd^ February 2017. In the actual data collection exercise, two FGDs, one with fishermen (males) and another with fish preservers (females), were held. Each FGD consisted of twelve participants. It is worth stating that fishing and fish preservation were male- and female-dominated activities, respectively. The rationale for the gender-specific FGDs and interviews was in recognition of the finding in the literature [18] that both males and females have roles to play in sanitation management at the community level, although at the household level women are expected to do more in the African context. In addition to the FGDs, in-depth interviews were held with three individual fishermen, three individual fish preservers, two leaders of fishermen association, and two leaders of fish preservers association. Furthermore, data were gathered with in-depth interview guides from three experts (an environmental health officer, a fisheries and aquatic sciences expert, and a water and sanitation expert). All respondents were purposively selected. All interviews were conducted face-to-face except the one with the water and sanitation expert, which happened on the phone because the interviewee failed to honour three earlier scheduled face-to-face interviews.

For ethical reasons, the objectives of the study were explained to the prospective respondents. Only those who freely agreed to participate as respondents were involved in the study. Participants were assured of confidentiality concerning information they would provide and anonymity regarding nondisclosure of their identity in the report. The literate respondents read and signed consent forms to show their agreement to participate voluntarily. The illiterate participants gave verbal consent and thumb-printed the informed consent forms after the content of the form had been translated into their local language for them to understand. The translation, agreement, and signing of the consent for the illiterate participants happened in the presence of witnesses who also signed. Data were digitally recorded with the permission of the respondents, and where permission for recording was not granted, hand-written notes were taken. Observations were done with a checklist and field notes taken.

Data saturation was considered and ensured. Seale [19] has argued that there is no one-size-fits-all method to reach data or theoretical saturation. This is because study designs are not universal. However, researchers do agree on some general principles and concepts concerning theoretical saturation, including no new data in terms of data collection, no codes/coding, and no new themes in terms of data analysis [20]. When and how one reaches those levels of saturation will vary from one study design to another. Guided by this, a pragmatic approach was followed to reach theoretical saturation. That is, data collection and analysis ceased when it was realized that continuing with collection or analysis produced no additional meaningful results because all that was needful had been exhausted. Sampling and data collection continued until no new conceptual insights were generated. In other words, theoretical saturation occurred when the researcher and research assistants saw that nothing new was being discovered from further interviews and focus group discussions. This saturation point was reached having conducted two FGDs and thirteen IDIs from a total of 37 participants. With the data from this number of participants, it was realized that all that was needed to address the objective of the study had been obtained, so there was no need to conduct more FGDs and interviews as it would be superfluous to do so. In fact, for similar questions across some of the instruments, similar responses were being obtained at the point of saturation, making it unnecessary to collect additional data. Attainment of data saturation shored up the external validity of the instrument and the data collection processes.

The qualitative data were transcribed and analysed manually by the researcher and the three research assistants. In the first phase, open coding of the interview transcripts was done, which led to identifying a set of recurring themes, that is, first-order concepts in the data. In the second phase, the related first-order concepts were merged which led to higher-order categories of aggregate dimensions. In the third analysis phase, the data were put into theoretical categories to create a “data structure” representing the findings. These recursive and iterative analytical processes continued until a theoretical saturation point was reached. This was the point where no new codes and themes were emerging any longer, suggesting that the analysis was optimum. At each stage of the analysis, extensive discussions took place among the researcher and research assistants. Any disagreements at each stage were resolved before proceeding to the next. The presentation of findings was guided by the overarching objective of the study and done in the context of the literature reviewed. The thick descriptive narrative mode of interpretation was used to present the results. Most significant stories and noteworthy quotations from the transcripts were used to highlight major arguments in the findings.

## 3. Results and Discussion

The results and discussion are presented under five broad themes, namely, respondents' perception of waste, fisherfolk's attitude to waste disposal, implications for health, implications for aquatic resources, and implications for sustainable development.

### 3.1. Respondents' Perception of Waste and Waste Disposal

As noted in the literature review, perception plays a major role in waste disposal practices. If people have negative perceptions about waste, they will pay no or little attention to its proper disposal and vice versa. Therefore, setting the tone for the primary data collection, respondents were asked to express their thoughts about waste as a concept. This was in line with Alam and Ahmade [21] argument that defining the concept of waste is the foundation of understanding and managing waste properly. In response to the question regarding the definition of waste, the concept was described variously by the various categories of respondents. Typical descriptions as abstracted from respondents' perception of waste are presented below.

*Waste is a material that is abandoned because it is not wanted (a 54-year-old fish preserver)*.

Waste is what remains to be disposed of when you select and/or use the good part of the item(s) available to you (a 51-year-old fisherman).

*It is anything thrown away or abandoned because it will not serve any useful purpose (a 44-year male FGD discussant ****).***

Contrastingly, the Environmental Health Expert had this to say about the concept of waste.

*“Waste is a discarded material which has no consumption value to the entity abandoning it. It could be in the form of solid, liquid, or gas. The gaseous wastes are principally industrial fumes and smoke; while the liquid wastes consist mainly of sewage and the fluid part of industrial wastes. Solid wastes, on the other hand, are often classified as refuse. However, it should be noted that waste is a relative concept because what is waste to one person can be a resource to another person, and what is a waste at one place or time can be a resource at another place or time.*”

It was gathered from the descriptions by the various respondents that there were similarities and differences between the fisherfolk's and the expert's perceptions of waste. The similarity was that both the expert and the fisherfolk primarily perceived waste as an unwanted and discarded substance or material. However, the expert's description of waste was more scholarly, academic, and detailed than the fisherfolk's as it went further to identify types of waste to make it clearer. The other remarkable difference was that the expert saw waste as a possible resource but that was not evident from the fisherfolk's perception of waste. However, these perceptual differences were expected due to the differences in the levels of education between the fisherfolk and the expert. That is, while the expert was learned or highly formally educated and, therefore, looked at the issue from a broader perspective, the fisherfolk were unlettered; therefore, they were narrow in terms of perspective. This is consistent with Shah and Shah's finding [22] that the level of education plays a major role in the perception of and attitude to waste disposal, while it contradicts Sessa et al.'s [23] conclusion from their study in Italy that education does not necessarily influence perception regarding solid waste disposal.

Furthermore, the fisherfolk were asked to identify who in their opinion should be responsible for proper solid waste management in the fishing community. The key responsible entities they identified were the central government, municipal assembly/authority, traditional authority, Member of Parliament for the Constituency, Zoomlion Ghana Limited, local assembly members, and the fisherfolk themselves. The fisherfolk were then asked to indicate, by ranking, who should be held responsible for ensuring that waste was properly managed in the community.  Table 2 shows fisherfolk's responses regarding the responsibility for managing waste generated at the fisherfolk's work environment. It would be recalled from the methodology section that male represents fishermen, while female represents fish preservers or processors.

The table shows that the municipal authority is ranked first by the fisherfolk (both male and female) in terms of responsibility for ensuring proper sanitation in the community including where the fisherfolk plied their trade near the lagoon and the sea. Concerning the same issue, Boadi [24] found that 46% of the respondents expressed the opinion that the onus lies on the Cape Coast Metropolitan Authority, Ghana, to ensure a clean environment.

Zoomlion Ghana Limited was also ranked high, that is, second and third, respectively, by the male and female fisherfolk because the fisherfolk perceived that this was their work since they were paid to clean the streets. Interestingly, the male and female fisherfolk perceived that they (themselves) came third and fifth, respectively, regarding the responsibility to keep the fishing environment clean. Though this showed the fisherfolk acknowledged that they had a role to play in maintaining solid waste in the community, they did not perceive themselves as the primary or foremost duty bearers in the solid waste management endeavour.

It is also seen from Table 1 that the females ranked the central government high (i.e., second) as far as responsibility for sanitation in the community was concerned. The reason given by the females (fish preservers) for ranking the central government second was that it was the central government that should impress upon the local government (municipal assembly) to enforce the environmental sanitation laws because the government is a higher authority, although the municipal authority has the primary responsibility concerning sanitation management at the community level. Both male and female fisherfolk maintained that they paid tax to the government and municipal assembly through the Ghana Revenue Authority and the municipal authority's tax collectors; therefore, part of the tax should be used to clean the fish landing site.

Some of the fisherfolk felt that the traditional authorities were the custodians of the land and they commanded respect, so they could make sure that indiscriminate waste disposal practices did not occur in the community, including areas where the fisherfolk plied their trade. Other fisherfolk were of the view that the local assembly members were the ones who had been voted for to spearhead community development, including sanitation management, so they should spearhead the maintenance of proper waste disposal standards at the fish landing site, which was part of their (assembly members') jurisdiction.

Other reasons adduced by the fisherfolk for assigning responsibilities for solid waste management bordered on politics and social contract. In that regard, some fisherfolk pointed out that whenever the political parties and even local assembly members are seeking political power, they make lots of promises including ensuring proper sanitation, but when they are elected, they forget about their promises. They maintained, for example, that the current government of Ghana promised, when they were in opposition, that they would make Accra, the capital of Ghana, the cleanest city in Africa by the end of their first term (4 years ending December 2020) of office, but from the stories they heard and the pictures they saw on television, it was clear that Accra was still grappling with sanitation challenges although the government was in its fourth year and was about to end its term of office. They added that the previous governments said a lot about sanitation but there was no remarkable improvement in sanitation at the end of its term. According to one female participant,

*Accra and the cities are not the only places in Ghana where people live. This place is also part of Ghana*, *so the government should make sure the place is clean. We know we also have to do our part*, *but the government should lead the Crusade by providing facilities, enforcing the laws, and doing more sensitisation on sanitation. Politicians and Governments should honour their social contracts with the people. We are tired of promises that are never fulfilled. The politicians are all the same. They promise a lot and do very little.*

The above evidence supports Melissa's [25] and Oyedotun et al.'s [10] views about the relevance of perception for waste disposal. Melissa [25] sees perception as a way of understanding or thinking about something while Oyedotun et al. [10] argue that perception relates to the state of being aware of something through the senses—sight, hearing, taste, smell, and touch feeling. The linkage is that the fisherfolk hear what politicians say when they are seeking votes for political power and this influences their perception of politicians' role in managing the waste disposal menace in the community. These show that perception could be useful in waste disposal management as it provides insights into the issues and helps to analyse them for effective solutions.

### 3.2. Fisherfolk's Attitude to Waste Disposal

Three main issues were addressed as regards fisherfolk's attitude to waste disposal. These were (1) demand for waste collection services as demonstrated in their willingness to pay for waste collection services, (2) waste segregation, and (3) where solid waste was disposed of.

Akkajitet al. [26] suggest that people's attitudes influence their behaviour towards waste disposal, for example, in terms of demand for waste collection services. This was verified with respect to willingness to pay for solid waste collection services. The fisherfolk were asked if they were willing to pay for waste collection services. The dominant responses from both the fishermen and fish preservers indicated that they were not. The reasons for their unwillingness to do so were mainly poverty-related. In the words of most of them,

“*Our fishing business is seasonal, making our incomes not only low but also irregular. We are already saddled with heavy economic burdens due to payment of school fees, funeral costs, and religious and family obligations, so we cannot pay for waste disposal also. We pay for the “useful,” not the “waste”. We pay tax so the government should help us with such services*.”

A few others, however, indicated that if the services would be effective and efficient, they would be prepared to pay a token for the waste collection services. From literature, modern best practices in solid waste disposal require that waste be sorted before final disposal. In the past, solid waste was just dumped into a waste receptacle to be carted away by the municipal garbage collectors. The attitude regarding waste disposal of the old generation is no longer acceptable and is giving way to the modern practices where wastes are sorted due to heightened awareness, consciousness, and enlightenment. In most developed and some developing countries today, garbage is segregated according to components that can be recycled and those which cannot be re-used such as the organic and perishable wastes that would have to be disposed of properly because they will decompose and become putrid [18].

As part of the investigation into the fisherfolk's attitude to waste disposal, they were asked if they sorted their solid waste for disposal. It emerged that none of them sorted their waste. While most of the fisherfolk did not see the need for waste segregation, others said separate bins had not been provided by the municipal assembly and/or Zoomlion for sorting of waste, so they dumped all manner of solid waste into the same bin. This attitude was found to be at variance with modern best practices of waste disposal. Observation revealed that the greater part of the waste generated by the fisherfolk at their workplaces (fish landing and preservation sites) included metal cans, plastic products including cans and bottles, polythene bags, discarded canoes, abandoned fishing gears, discarded freezers, food materials, wood, and papers.

**Figure 2:**
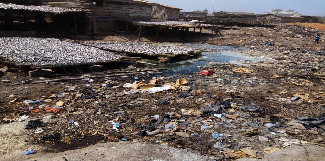
Solid waste disposed onto the street at fish preservers' workplace (source: field data, 2017).

On where they disposed of their solid waste, most of the fisherfolk mentioned waste bins provided by the municipal assembly, but a few confessed that they sometimes did so at the open dumpsites, into the drains, lagoon, sea, or onto the street (see Figure 2). None of the fisherfolk mentioned other disposal methods such as incineration, composting, or recycling. It also became evident that many of the fisherfolk were expecting the municipal authorities to provide more bins at vantage points for the collection of solid waste. Where the bins had not been provided or where the bins provided were not close, some fisherfolk disposed of the solid waste anywhere (onto the street, into gutters, the lagoon, or the sea) A fish preserver had this to say in connection with the disposal habit*:*

“*I must confess that for empty water sachet and other wrappers, I throw them onto the street*, *but if there is a bin close by, I put it into it. The Zoomlion workers tidy up the place every day. It is their work. They are paid for it. That is how they also earn their income. Many people even say that if we do not litter, they will have no work to do. Although that perception is unfortunate, it is a fact.*”

Another fisherfolk, supported by a few others, opined that *“We have spoken about the provision of more waste bins at vantage points to enable us to dispose of solid waste into them for collection and final disposal by the municipal assembly. Anyway, the municipal assembly and Zoomlion have done well by providing some waste bins at some vantage points*, *but the bins are not enough. In some cases, the bins are far from some of us. Those close to the bins dispose of the garbage into them but where the bins are far away, we are discouraged*, *so some people sometimes dispose of the waste onto the street, into drains/gutters, the lagoon, or the sea. Most people will deny this*, *but it is real” (FGD, fishermen*).

**Figure 3:**
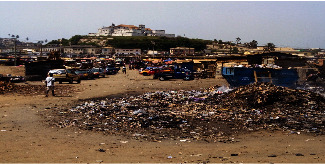
An overflown solid waste container at the fish landing/preservation site (source: field data, 2017).

Similarly, the common reports from the female FGD on the same issue are summarised as follows:

*When we dispose of the waste into the bins and they are full, the municipal assembly and Zoomlion will not come over to evacuate it in time; therefore, scavengers such as the vultures and crows as well as flies will be disturbing us due to the overflown containers (see*Figure 3*). With this, is it not better to dispose of the waste straight into the lagoon or the sea so it will be carried away straightaway to save us from the filthy environment and its associated dangers of diseases? The assembly should be blamed for our poor attitude to waste disposal (FGD, fish preservers)*.

The evidence supports Kwon and Silva's [27] observation about human behaviour, which is guided by three key issues. These are beliefs about the likely outcomes of the behaviour and the evaluations of these outcomes (behavioural beliefs), beliefs about the normative expectations of others and motivation to comply with the expectations (normative beliefs), and beliefs about the presence of factors that may promote or hinder the performance of the behaviour (control beliefs).

It is gathered from the responses that the factors responsible for fisherfolk's poor attitude to waste disposal were inadequate bins provided by the municipal authority, bins not close or within immediate reach, and irregular evacuation of filled bins. With these factors, the motivation to comply with the acceptable standard of disposing of waste into the available bins is reduced. Also, in the quotes above, the fish preservers believed that disposing of the waste straight into the lagoon or the sea will save them the unclean environment and its attendant risks of diseases, hence the motivation to dump waste into the water bodies.

Additionally, some of the fisherfolk perceived that “*no litter means no job*” for the Zoomlion staff since their duty was to ensure a clean community environment. These perceptive factors triggered negative attitudes, which translated into unapproved disposal behaviour and practices. This supports the evidence from the literature which suggests that perception and attitude are influenced by one's knowledge, beliefs, values, norms, and culture, all of which conspire to explain waste disposal behaviour and practices. In short, what these imply is that the more positive one's perception of and attitude to waste disposal are, the more likely one will put up positive behaviour and practice in terms of waste disposal and handling.

### 3.3. Implications for Health

Ziraba et al. [4] described improper waste disposal as a potential threat to public and environmental health. In order to determine if the fisherfolk were aware of the health implications of indiscriminate disposal practices, they were asked to state the implication of poor waste disposal practices for human health. The fisherfolk stated that improper disposal of waste on land and into the lagoon polluted both the water bodies and air, making the place smell bad which was not good for both human and environmental health. This evidence was consistent with Aglanu and Odame Appiah's [28] finding that as a result of waste disposed into the Korle Lagoon in the Greater Accra Region of Ghana, stench wafts back from the lagoon to the adjoining town that is home to hundreds of families.

All the fisherfolk indicated that waste disposed into the environment could cause diseases such as malaria and cholera. This not only supported Boadi's [24] finding in a study in Cape Coast Metropolis but also showed that the fisherfolk were aware that poor waste disposal practices had negative health and livelihood implications. The fisheries and aquatic sciences expert indicated that there had been instances where amounts of fine plastic particles were found in fishes and that these plastic particles could end up in human bodies once the fish was consumed, thereby causing health problems to the unsuspecting consumers(s). The environmental health officer said

*The health implications include allergic rhinitis, asthma, chronic bronchitis, skin disorders, fungal infection, allergic dermatitis, pruritis, and skin cancer; respiratory abnormalities such as upper respiratory tract infections; and other health challenges related to sanitation. Indiscriminate waste disposal practices by the fisherfolk also cause visual and air pollution. The trash on the beaches and in the water bodies downgrades the living standards of the people as it is aesthetically repugnant and offensive*.

The quote is consistent with Ahmed and Isaac's [5] finding that some of the consequences of improper waste disposal in and around the Keta Lagoon in Ghana were water and air pollution, floods, and health issues. It is clear from the pieces of evidence that all categories of respondents were aware that poor waste disposal practices had negative implications for public and environmental health. However, as expected, the expert had more knowledge about the health implication of the phenomenon than the fisherfolk. This means that the experts could be used to educate the fisherfolk more on the health implications of poor waste disposal practices, using the fisherfolk's level of awareness as the starting point and building on it.

### 3.4. Implications for Aquatic Resources

Poor solid waste disposal practices could have several consequences for water resources. Questions were posed to respondents to elicit their perceptions of the implications of solid waste disposal practices for aquatic resources. The fisheries and aquatic expert indicated that

*“Some species of fish may become endangered or even go extinct as a result of pollution through dumping of waste. For instance, if a specific species of a turtle only occurs in a small ocean area, and the area becomes polluted through dumping of waste, this turtle species may go extinct since its natural habitat has been destroyed. Furthermore, when the waste gets into the water bodies in large quantities, it could impede navigation.*”

The finding that improper waste disposal practices could impede navigation corroborated that of Ahmed and Isaac [5]. According to Ahmed and Isaac, fisherfolk in the Keta community in Ghana reported that waste in the Keta Lagoon was creating navigational challenges for the fishermen. The fisherfolk expressed mixed perceptions about the implications of waste disposal for aquatic resources. Some fisherfolk maintained that when waste got into the sea, it (the sea) brought the unwanted materials back during high tide. According to one fisherman:

….. *the sea is a god. It does not want dirty things*, *so unclean materials that get into it are often washed ashore; therefore*, *it cannot usually have a serious effect on the aquatic resource. If you take a walk along the beach now, you will see some wastes washed ashore by the sea…..*

Another fish preserver indicated that part of the waste is a source of livelihood to the fish because the fishes feed on it. According to the 59-year-old fisherman, “*the fish eat faeces and food particles or left-overs that get into the lagoon and sea. It is the gravels, plastics, metals, and pieces of wood that the fishes do not like*.”

These quotes show that cultural beliefs are also responsible for poor waste disposal attitudes and practices. Another fish preserver opined that improper waste disposal practices in the area could lead to a decline in fish population which could ruin fish-related livelihoods. According to the fish preserver, if the fish population reduced, fishermen might not be able to catch enough fish for their consumption and sale and might be forced to relocate or migrate to other places for sustainable livelihoods. A fisherman argued that waste that got into the water bodies as a result of his waste disposal practices was insignificant given the size and depth of the sea and lagoon. He stated that “*many people have done the same for centuries*, *but the sea and the lagoon are still as accommodating and resilient as ever. My small amount of waste cannot have a significant effect on these big water bodies although I do not mean we should continue to dump waste into them*.”

It can be seen from the above quote that some of the fisherfolk failed to recognise that just as little drops of water make the mighty ocean, the small amounts of solid waste that get into the water bodies aggregate to pose challenges to the aquatic resources. While similarly mixed responses were distilled from the FGDs with the fish preservers and fishermen, the experts made different submissions regarding the impact of solid waste disposal on aquatic resources. Contrary to the fisherfolk's perception that the fish fed on the wastes, the fisheries expert submitted that most wastes were rather a nuisance to the fish as the fishes were more comfortable in their natural habitat without the waste; besides, the waste could kill the fish if the waste was toxic. According to the expert, some marine species were known to be harmed or killed by plastic debris. Marine animals are mostly affected through entanglement in and ingestion of plastic litter. Furthermore, abandoned fishing gear caused ecological concerns. The expert reported that most of the materials used in fisheries were polyethylene, polyamide, polypropylene, polyester, and other synthetic materials that were mostly nonbiodegradable. The expert revealed that the lifetime of plastic materials in the marine environment varied depending on the environmental conditions and may extend to hundreds of years for complete mineralization. These reports were consistent with Boopendranath's [2] and Andrady's [29] findings on similar issues. The implication is that the increasing plastic materials in seawater and marine sediments could pose a long-term threat to the fisheries and aquatic resources. The fisheries and aquatic scientist observed that

*Oceans are complex ecosystems where water organisms depend on each other, and some of the organisms are fragile. If the populations of some aquatic species decrease due to water pollution resulting from the dumping of waste into the water bodies, other aquatic animals that depend on them for survival may also suffer since their sources of food may be depleted. This implies that the fish population is likely to drop in the affected areas. This may lead to marine ecosystem imbalance. Due to anthropogenic activities including poor waste disposal practices, the lagoons and the sea are already enduring a high concentration of metallic and plastic wastes which are unhealthy to the aquatic ecosystem*.

**Figure 4:**
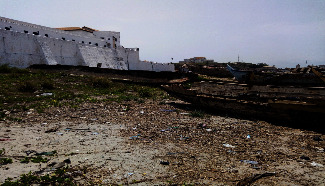
Abandoned canoes near Elmina Castle, the Benya Lagoon, and the sea (source: field data, 2017).

The water and sanitation expert as well as the fisheries and aquatic scientist maintained that a significant amount of waste created in the process of fishing-related activities (navigation on the water bodies and use of fishing boats, canoes, nets, fishing gear, metal cans, plastic, and paper products) such as derelict fishing gear, abandoned boats, discarded canoes (see Figure 4), by-catch discards, and plastic wastes posed challenges to fishing activities in particular and the marine environment in general. Similarly, activities of the fish preservers, such as artisanal fish smoking, drying, salting, and freezing, were associated with waste generation, part of which eventually ended up in the marine environment, posing health and aquatic challenges.

The water and aquatic scientist further reported that indiscriminate waste disposal in the fishing environment could lead to loss of aquatic biodiversity. According to the expert, sea organisms such as coral reefs were sensitive to changes in living conditions; therefore, a severe adverse environmental change resulting from the dumping of waste could kill them. These pieces of evidence support Karikari et al.'s [6] finding, as cited in Aglanu and Odame Appiah [28], that due to high level of pollution resulting from improper waste disposal, many organisms have been unable to survive in the Korle Lagoon in Ghana for years. In sum, while the fisherfolk had mixed perspectives about the effects of solid waste disposal on aquatic animals, the expert was clear that the wastes were a nuisance to the animals and had negative effects on them when the water bodies acted as a sink for the anthropogenic pollutants.

### 3.5. Implications for Sustainable Development and the Way Forward

Goal 6.2 of the SDGs enjoins all stakeholders to ensure universal access to adequate clean water, equitable sanitation, and hygiene and end open defecation by 2030. Since SD is generally considered to have three dimensions, namely, environmental, economic, and social sustainability, the responses from the participants were analysed in terms of their implications for these dimensions, which are seen as the pillars of SD.

Regarding environmental sustainability, the analyses of the responses showed that improper waste disposal attitudes led to practices that degraded the environment and polluted the lagoon and the sea, resulting in damage to or loss of biodiversity. As far as economic sustainability is concerned, the fisherfolk's attitude to waste disposal could reduce the contribution of the fishing industry to GDP through less employment of factors of production. This was because once there was less fish harvest resulting from the reduction in the fish population, people whose livelihoods depended on the fishing industry could lose their livelihoods. In terms of social sustainability, the practices could pollute the environment (air, land, and water bodies) and cause public health problems. Sicknesses resulting from improper waste disposal by the fisherfolk could generate health problems which could have socioeconomic implications for the local people and others that may come in contact with the polluted environment.

When asked about the implications of the fisherfolk's attitude to waste disposal for sustainable development, the three experts (water and sanitation expert, fisheries and aquatic scientist, and environmental health expert) shared similar views as summarised below:

In general, fishing-related activities create a variety of litter. Unsustainable practices of fishing, fish preservation, and processing can be a source of litter such as plastic bottles and bags, aluminum cans, paper bags, metals, and leftover food waste, discarded by-catch in the lagoon, the sea, and on land. If the indiscriminate waste disposal activities of the fisherfolk are not regulated, they could cause serious problems for maintaining the marine and coastal environments. It might be thought that these fisherfolk's waste disposal practices constitute a small threat to the environment but as they do it every day and others do similar things elsewhere, the wastes accumulate and create a big local challenge, graduating to a national challenge and eventually to a global challenge. In these circumstances, the health of the people, the ocean, and the lagoon could be compromised, while marine wildlife and ecosystems could also be unsafe. These public health and ecological challenges could threaten the livelihoods of the fisherfolk and reduce the GDP of the country. What should be noted is that these negative environmental, economic, and social effects will impact not only the livelihoods of the present generation but the future generations as well.

The experts' views on the fisherfolk's waste disposal practices suggested that the practices had implications for SD. It is clear from the summarised responses that just as the effects of the seemingly “small” local actions and/or inactions cumulatively become a global problem, achieving SD also starts with “small” local actions that are taken individually or by “small” groups on a daily and/or regular basis. It is these “little” actions and/or inactions that eventually help or mar the achievement of the global SD targets and goals. Given the confluence of the experts' views as summarised in the above quote, it can be argued that although the SD agenda is global in orientation, it is local in its true sense. It also suggests that SDG (14) on sustainable use of the oceans, seas, and marine resources and SDG (6) on water and sanitation are compatible and mutually supportive. This implies that nonachievement of the sanitation goal would pose challenges for the sustainability of marine resources, which will impact the associated livelihoods presently and in the future.

On the way forward, the fisherfolk suggested that the government should provide adequate infrastructure for waste disposal and enforce the sanitation laws. The experts, on their part, opined that the more likely the fisherfolk perceived that their waste disposal attitudes could affect sustainable health, environment, and aquatic resources, the more likely they would engage in environmentally friendly behaviours and practices; therefore, they need to be educated and equipped with proper waste disposal knowledge and practices. Further, the experts observed that since some people were likely to continue to engage in indiscriminate waste disposal practices despite the sensitisation, the fisherfolk's waste disposal practices should be regulated and monitored to ensure that they comply with best practices. One key issue that was prominent in both the fisherfolk's and the experts' views on the way forward was regulation. This implied that the issue of law enforcement and monitoring needed to be taken seriously by the authorities to ensure that the fisherfolk adhered to acceptable waste disposal practices in their workplace environment for SD.

## 4. Conclusion

The study examined the perception and attitude of fisherfolk regarding solid waste disposal in a coastal fishing community in Ghana and the implications of these for public health, aquatic resources, and sustainable development. It emerged, as expected, that the experts had a deeper and broader conception of waste than the fisherfolk. Unlike the fisherfolk who perceived waste as a nuisance and useless, the experts perceived it as a nuisance and a possible resource. The fisherfolk did not segregate their solid wastes in line with best practices. Also, citing poverty as the main reasons, they were unhappy about payment for waste collection services.

While the fisherfolk acknowledged that they had a role to play in ensuring proper solid waste disposal in their environment, they expected the municipal authority, central government, and private sanitation companies (Zoomlion) to play a key role in managing solid waste in the community. This position was informed by their opinion that since they paid their taxes, part of the tax revenue should be used to take care of sanitation in their workplace environment. The fisherfolk were aware that poor waste disposal practices could cause diseases such as malaria and cholera, but their perceptions of the effects of their waste disposal attitude on aquatic resources were mixed. While the experts maintained that improper waste disposal could harm marine biodiversity since many sea organisms were sensitive to changes in their living conditions, some of the fisherfolk perceived the sea as resilient, arguing it had acted as a resilient sink for waste dumped into it since time immemorial.

In addition to the common sanitation and hygiene-related diseases (malaria and cholera) that were known to the fisherfolk, the experts identified several other public health implications of improper sanitation including fungal infection, allergic dermatitis, skin cancer, and respiratory abnormalities. Besides, the experts observed that the waste created through fishing-related activities such as derelict fishing gear, abandoned boats, discarded canoes, by-catch discards, and metallic and plastic wastes posed challenges to fishing activities in particular and the marine environment in general.

The fisherfolk's attitude to waste disposal had implications for SD. The attitude led to practices that affected social sustainability through their public health implications and negative effects on fishing-associated employment opportunities. The practices affected economic sustainability. The waste had the potential to reduce the fish population, leading to less fish-catch, which had a negative effect on the local economic development and livelihoods. The practice affected environmental sustainability as it degraded the physical environment and polluted the water bodies (lagoon and the sea). This could affect not only the aquatic ecosystems for only the present generation but also the future generation. The government and municipal authority should collaborate with the experts in environmental health, water and sanitation, fisheries and aquatic sciences, and traditional authorities to sensitise the fisherfolk on the sustainability implications of unapproved solid waste disposal practices and best practices regarding the issue. The government and municipal authority should also provide more waste disposal infrastructure and enforce the laws to change fisherfolk's perception of and attitude to waste disposal to ensure the maintenance of best practice for sustainable development.

## 5. Limitation and Suggestion for Future Research

The study employed the qualitative case study design, using relatively few respondents. This limited the generalisation of the results. Other studies can approach it from quantitative design using many more communities/households along with the coastal areas of Ghana and comparing the results. Again, the study looked at the disposal of solid waste only. Other studies could look at disposal of liquid waste since this aspect of waste management at the household and community levels appears to be under-researched. Furthermore, other studies could look into managing fisherfolk's solid waste disposal attitude, behaviour, and practices at the national and international levels.

## Figures and Tables

**Table 1:** Profile of respondents, data collection approach, and instruments used.

Respondents	Male	Female	Total	Approach	Instrument
Environmental health officer	1	0	1	IDI	IDI guide
Fisheries and aquatic sciences expert	1	0	1	IDI	IDI guide
Water and sanitation expert	1	0	1	IDI	IDI guide
Fishermen association leaders	2	0	2	IDI	IDI guide
Fish preservers association leaders		2	2	IDI	IDI guide
Fish preservers/processors (individuals)	3	3	6	IDI	IDI guide
Fish preservers/processors (group)	0	12	12	FGD	FGD guide
Fishermen (group)	12	0	12	FGD	FGD guide
Total	20	17	37	NA	NA

Note: FGD = focus group discussion, IDI = in-depth interview, NA = not applicable (source: the author's construction based on respondents' characteristics and data collection methods).

**Table 2:** Fisherfolk's ranking of key stakeholders in terms of responsibility for ensuring proper sanitation in the community.

Responsible entity	Ranking by males	Ranking by females
Municipal authority/assembly	1^st^	1^st^
Zoomlion Ghana Limited	2^nd^	3^rd^
Fisherfolk	3^rd^	5^th^
Central government	4^th^	2^nd^
Traditional authority (community chiefs and elders)	5^th^	6^th^
Assemblymen/women (elected local representative)	6^th^	4^th^
Member of Parliament (elected national representative)	7^th^	7^th^
Others	8^th^	8^th^

Source: field data, 2017. Note: males = fishermen; females = fish preservers.

## Data Availability

The data used to support the findings of this study are included within the article.
